# Melatonin Attenuates Heat Stress-Induced Metabolic Labeling Remodeling in Primary Goat Sertoli Cells

**DOI:** 10.3390/vetsci13070678

**Published:** 2026-07-13

**Authors:** Guang Yang, Pengyun Ji, Lu Zhang, Zhou Yu, Guoshi Liu

**Affiliations:** 1 College of Animal Science and Technology, Sanya Institute of China Agricultural University, Sanya 572025, China; yangguangimu@163.com (G.Y.); jipengyun@cau.edu.cn (P.J.); luzhang2018@cau.edu.cn (L.Z.); 2 State Key Laboratory of Farm Animal Biotech Breeding, Frontiers Science Center for Molecular Design Breeding, College of Animal Science and Technology, China Agricultural University, Beijing 100193, China

**Keywords:** extracellular acidification rate, glutamine tracing, goat, heat stress, melatonin, reductive carboxylation, Sertoli cells, tricarboxylic acid cycle

## Abstract

Heat stress impairs male reproductive function, but whether acute heat stress alters glutamine-derived carbon labeling in Sertoli cells and whether melatonin modulates this response remain unclear. This study aimed to determine whether acute heat stress alters glutamine-derived carbon labeling in primary goat Sertoli cells and to evaluate whether melatonin attenuates these heat stress-associated metabolic changes. Primary goat Sertoli cells were assigned to control, heat-stress, or heat-stress plus melatonin groups. Cells were labeled for 24 h with [U-^13^C_5_] glutamine. Cells in the heat-stress and heat-stress plus melatonin groups were subsequently exposed to 42 °C for 0.5 h, whereas control cells were maintained at 37 °C. In the heat-stress plus melatonin group, melatonin (0.5 μM) was applied throughout labeling and heat exposure. GC–MS was used to measure mass isotopologue distributions, which were subsequently corrected for natural isotope abundance, and to calculate total ^13^C-labeled fractions of selected TCA cycle intermediates; extracellular acidification rate (ECAR)-derived parameters were measured as indirect indices of glycolysis-associated acidification. Heat stress increased the total ^13^C-labeled fraction of cis-aconitate and, as an exploratory combined readout, the unweighted average total ^13^C-labeled fraction of citrate and cis-aconitate; succinate and malate were unchanged, while fumarate decreased. Melatonin reduced the heat stress-associated total ^13^C-labeled fractions of citrate and cis-aconitate and decreased the relative abundance of cis-aconitate M + 5. Melatonin also attenuated heat stress-associated increases in ECAR-derived glycolysis, glycolytic capacity, and glycolytic reserve. These findings indicate that acute heat stress is associated with altered citrate/cis-aconitate ^13^C-labeling patterns that are potentially compatible with reductive carboxylation-related labeling but do not directly demonstrate altered pathway flux; these changes were attenuated by melatonin. Overall, the study objective was achieved by showing that acute heat stress altered citrate/cis-aconitate ^13^C-labeling patterns and increased ECAR-derived extracellular acidification in primary goat Sertoli cells, and that melatonin attenuated both responses. Further studies incorporating a melatonin-only group, dynamic isotope tracing, mitochondrial respiration measurements, redox analysis, and flux modeling are warranted to define the underlying metabolic routes more directly.

## Simple Summary

Heat stress impairs male fertility in farm animals, but the Sertoli cell metabolic responses underlying this effect remain incompletely understood. Sertoli cells are testicular support cells that provide nutrients and help maintain a stable environment for developing sperm. This study examined whether acute heat stress alters glutamine-derived carbon labeling in primary goat Sertoli cells and whether melatonin attenuates these changes. Glutamine is an important carbon and nitrogen source that contributes to cellular energy metabolism and biosynthesis. We found that heat stress altered glutamine-derived ^13^C-labeling patterns in selected TCA cycle intermediates and increased ECAR-derived extracellular acidification associated with glycolysis-related metabolism. Melatonin treatment attenuated these heat-related metabolic changes. This work provides a cell-based metabolic perspective that may help future studies investigate how elevated temperature affects male reproduction in livestock.

## 1. Introduction

Environmental heat stress is a major factor that compromises livestock productivity and reproductive performance [1]. The male reproductive system is particularly sensitive to elevated temperature, and thermal stress can impair spermatogenesis, disturb testicular redox homeostasis, and reduce sperm quality [2]. Sertoli cells are the major somatic cells within the seminiferous epithelium and provide structural, nutritional, and metabolic support for developing germ cells [3]. Because germ cell development depends strongly on Sertoli cell metabolic support, altered Sertoli cell metabolism may represent one mechanism linking heat stress to impaired male fertility.

Beyond oxidative damage, apoptosis, and inflammatory activation, heat stress can also induce metabolic remodeling [4]. Such remodeling is increasingly recognized as an important component of cellular stress adaptation [5]. Sertoli cells coordinate glycolysis, the TCA cycle, amino acid metabolism, and mitochondrial energy production to maintain the metabolic environment required for spermatogenesis [6]. However, conventional steady-state metabolomics primarily provides information on metabolite abundance and, by itself, cannot directly resolve substrate-derived carbon redistribution within metabolic networks. Stable isotope tracing can provide additional information on substrate-derived labeling distributions and pathway-associated isotopologue patterns, although endpoint labeling data alone do not directly quantify metabolic flux [7].

Glutamine is an important anaplerotic substrate that contributes carbon to the tricarboxylic acid (TCA) cycle through glutamate and α-ketoglutarate [8]. After conversion to glutamate and α-ketoglutarate, glutamine-derived carbon can be incorporated into the oxidative branch of the TCA cycle, generating labeled succinate, fumarate, and malate. Alternatively, under conditions such as mitochondrial stress, altered redox balance, or constrained oxidative metabolism, glutamine-derived α-ketoglutarate may contribute to citrate and cis-aconitate isotopologue patterns compatible with reductive carboxylation-related labeling [9]. Thus, the distribution of [U-^13^C_5_] glutamine-derived labeling among TCA-related isotopologues can provide indirect information on heat stress-associated metabolic adaptation, although these endpoint readouts do not directly quantify pathway flux.

Melatonin is a pleiotropic indoleamine with antioxidant, anti-apoptotic, and mitochondria-protective properties [10]. Previous studies have shown that melatonin can alleviate heat stress-induced cellular injury in the male reproductive system [11]. Nevertheless, whether melatonin modulates nutrient-derived ^13^C labeling patterns in heat-stressed Sertoli cells remains unclear. In this context, most existing studies have focused on steady-state metabolite changes or downstream injury-related phenotypes, whereas the influence of melatonin on glutamine-derived ^13^C labeling redistribution under heat stress remains insufficiently defined [12]. Addressing this issue may help clarify whether melatonin modifies heat stress-associated metabolic labeling responses in thermally stressed Sertoli cells.

Therefore, this study used [U-^13^C_5_] glutamine stable isotope tracing to investigate the effect of acute heat stress on glutamine-derived ^13^C-labeling distribution in primary goat Sertoli cells and to determine whether melatonin modulates this response. By comparing oxidative TCA-associated readouts with citrate/cis-aconitate readouts potentially compatible with reductive carboxylation-related labeling, this work provides a cell-based metabolic perspective on how heat stress may remodel Sertoli cell metabolism. In addition, extracellular acidification rate (ECAR) was measured to evaluate whether changes in glutamine-derived labeling were accompanied by altered glycolysis-associated extracellular acidification. We hypothesized that acute heat stress would be associated with altered glutamine-derived ^13^C labeling in citrate/cis-aconitate readouts and that melatonin would attenuate this stress-associated labeling response. These findings may help clarify metabolic mechanisms underlying heat stress-associated impairment of male reproductive function and provide a basis for evaluating melatonin as a potential protective intervention.

## 2. Materials and Methods

### 2.1. Primary Goat Sertoli Cell Culture and Experimental Design

Primary goat Sertoli cells were isolated, purified, cultured, and characterized as previously described, and cell identity and purity were confirmed using Sertoli cell-specific markers before metabolic experiments [13]. The donor animals were healthy male Hainan Black goats aged 1.0–1.5 years with a body weight of 27.0 ± 3.4 kg (mean ± SD), obtained from Yingchi Family Farm, Yazhou District, Sanya, Hainan Province, China (109°07′28.08″ E, 18°21′43.10″ N). Before testicular tissue collection, all donor goats were confirmed to be in good physical condition and showed no obvious clinical signs of reproductive system disease or infectious disease. Testicular tissues were collected in March 2025 during the non-heat-stress season. Primary SCs used for the in vitro experiments were derived from three different donor goats, with each donor representing one independent isolation and culture batch and one biological replicate. All procedures involving animals were conducted in accordance with institutional guidelines and were approved by the Institutional Animal Care and Use Committee of China Agricultural University under approval number AW13014202-1-11.

Cells were maintained in DMEM/F12 (Servicebio, Wuhan, China) under standard culture conditions at 37 °C in a humidified atmosphere containing 5% CO_2_. When cells reached approximately 70–80% confluency, they were assigned to three treatments: control, heat stress, and heat stress plus melatonin. Cells in the control group were maintained at 37 °C for the corresponding treatment period. Cells in the heat stress group were exposed to 42 °C for 0.5 h as an acute heat stress treatment. In the heat stress plus melatonin group, cells were treated with 0.5 μM melatonin throughout the 24 h labeling period and during the subsequent 0.5 h heat exposure; this regimen is hereafter referred to as melatonin pre-/co-treatment. Melatonin-containing media were freshly prepared and protected from light. Melatonin (Sigma-Aldrich, St. Louis, MO, USA) was dissolved in DMSO (MedChemExpress, Shanghai, China) and added to the culture medium at a final concentration of 0.5 μM. Control and heat stress groups received an equivalent volume of DMSO alone, ensuring that the final DMSO concentration was identical across all groups and did not exceed 0.1% (*v*/*v*).

### 2.2. [U-^13^C_5_] Glutamine Stable Isotope Tracing

To examine glutamine-derived ^13^C-labeling distribution, a [U-^13^C_5_] glutamine (MedChemExpress, Shanghai, China) stable isotope tracing strategy was used. Glutamine tracing medium was prepared using glucose- and L-glutamine-free DMEM/F12 supplemented with 17.5 mmol/L unlabeled D-glucose, 2.5 mmol/L [U-^13^C_5_] glutamine, 10% dialyzed fetal bovine serum, and 1% penicillin-streptomycin. The glucose and glutamine concentrations were matched to those of the conventional DMEM/F12 formulation used for routine culture to minimize metabolic perturbation caused by altered substrate availability.

Cells were washed and then incubated with [U-^13^C_5_] glutamine-containing medium for 24 h. After the 24 h labeling incubation, the isotope-labeling medium was not replaced to avoid abrupt changes in substrate availability. The heat stress and heat stress plus melatonin groups were then transferred to 42 °C for 0.5 h, while the control group was maintained at 37 °C.

At the end of treatment, cellular metabolism was rapidly quenched to minimize post-treatment metabolic turnover. The culture medium was removed, and cells were washed twice with pre-cooled Dulbecco’s phosphate-buffered saline (DPBS). Metabolites were extracted using 80% methanol pre-chilled to −80 °C. Cells were scraped in pre-chilled extraction solvent on dry ice, transferred to pre-cooled microcentrifuge tubes, and then subjected to ultrasonic disruption on ice for 10 min at 200 W with 3 s pulses and 5 s intervals. The lysates were centrifuged at 14,000× *g* for 15 min at 4 °C. The supernatant was collected and dried under nitrogen. Dried extracts were stored at −80 °C until derivatization and gas chromatography–mass spectrometry analysis. Parallel samples from the same batch were used for protein quantification to monitor sample consistency. Raw mass isotopologue distributions were calculated from the relative peak areas of isotopologues within each selected metabolite-derived fragment, and corrected distributions were then obtained as described below. Mass isotopologue distributions were expressed as fractional abundances of each metabolite-derived fragment and therefore reflect relative ^13^C incorporation independent of total metabolite pool size; normalization to protein content was not performed for these fractional data. Parallel protein measurements confirmed that the protein content per sample did not differ significantly among groups, supporting the comparability of the reported isotopic distributions.

### 2.3. Derivatization and Gas Chromatography–Mass Spectrometry Analysis

Dried metabolite extracts were derivatized before gas chromatography–mass spectrometry analysis. Briefly, each dried sample was incubated with 50 μL of methoxyamine hydrochloride in pyridine (20 mg/mL) at 37 °C for 90 min. Subsequently, 50 μL of N,O-bis(trimethylsilyl)trifluoroacetamide with 1% trimethylchlorosilane was added, and the mixture was incubated at 70 °C for 60 min [14]. After cooling to room temperature, the supernatant was used for gas chromatography–mass spectrometry analysis.

Metabolites were analyzed using an Agilent 7890A gas chromatograph coupled to an Agilent 5975C mass spectrometer (Agilent Technologies, Santa Clara, CA, USA), with chromatographic separation performed on an HP-5MS capillary column (Agilent Technologies, Santa Clara, CA, USA). The initial oven temperature was set at 60 °C and held for 1 min, increased to 180 °C at 10 °C/min, then to 240 °C at 5 °C/min, and finally to 300 °C at 20 °C/min and held for 5 min. The inlet temperature was 280 °C, the ion source temperature was 230 °C, and the transfer line temperature was 280 °C. High-purity helium was used as the carrier gas at a flow rate of 1.0 mL/min. Each biological sample was analyzed as a single GC–MS injection. Analytical stability was monitored using pooled quality-control samples and solvent blanks throughout the analytical sequence. Electron impact ionization was performed at 70 eV, and data were acquired in full-scan mode over an m/z range of 50–600.

Raw data were processed using Agilent MassHunter Qualitative Analysis software (version B.07.00; Agilent Technologies, Santa Clara, CA, USA; https://www.agilent.com; accessed on 1 July 2026). Authentic standards for citrate, cis-aconitate, succinate, fumarate, malate, glutamine, glutamate, and aspartate were purchased from Sigma-Aldrich (St. Louis, MO, USA). Target metabolites were identified by comparison of retention times and characteristic fragment ions with these authentic standards. Peak areas of selected fragment ions were extracted for subsequent isotopologue analysis.

### 2.4. Isotopologue Distribution Correction and Calculation of ^13^C Labeling Indices

Mass isotopologue distributions were corrected for natural isotope abundance using a matrix-based correction method, and tracer isotope purity was incorporated according to the certificate of analysis for [U-^13^C_5_] glutamine [15]. Briefly, the theoretical natural isotope distribution matrix was constructed according to the elemental composition of the selected derivatized fragment ion for each metabolite. Mass isotopologue distributions were corrected for natural abundance contributions from metabolite backbone atoms and derivatization-derived atoms, including C, H, O, N, and Si, to obtain natural-abundance-adjusted distributions. The total ^13^C-labeled fraction was calculated as 1 − M + 0, representing the summed fractional abundance of all labeled isotopologues from M + 1 to M + n.

The ^13^C isotopic enrichment of each metabolite-derived fragment was calculated as [Σ(i × f_i_)/n] × 100, where i is the number of ^13^C atoms in the isotopologue, f_i_ is the corrected fractional abundance of the isotopologue with i ^13^C atoms expressed as a molar fraction, and n is the number of carbon atoms in the selected metabolite-derived fragment. For exploratory readout-level analysis, citrate and cis-aconitate were grouped as citrate/cis-aconitate total-labeled-fraction readouts, whereas succinate, fumarate, and malate were grouped as oxidative TCA-associated total-labeled-fraction readouts. The average total ^13^C-labeled fraction of each readout group was calculated as the arithmetic mean of the total ^13^C-labeled fractions of the corresponding metabolites and was used as an exploratory operational index for group-level comparison; this index was not weighted by metabolite pool size.

For isotopologue-specific interpretation, M + 5 isotopologues of citrate and cis-aconitate were considered operational readouts potentially compatible with reductive carboxylation-related labeling from [U-^13^C_5_] glutamine-derived α-ketoglutarate, whereas M + 4 isotopologues of succinate, fumarate, and malate were considered operational readouts of oxidative TCA-associated glutamine-derived labeling [16]. These total-labeled-fraction and isotopologue-specific indices were used to compare relative isotope-labeling distributions among groups and were not interpreted as absolute metabolic fluxes.

### 2.5. Extracellular Acidification Rate Analysis

The extracellular acidification rate (ECAR) was measured using a Seahorse XFe96 Extracellular Flux Analyzer (Agilent Technologies, Santa Clara, CA, USA). Under glycolysis stress test conditions, ECAR was used as an indirect readout primarily reflecting glycolysis-associated extracellular acidification [17]. Cells were seeded into Seahorse XF cell culture microplates at a density of 2 × 10^4^ cells/well in 100 μL of standard culture medium and cultured for 24 h to allow for attachment. Cells were then subjected to the same treatment regimens as in the tracing experiments (control, heat stress, or heat stress plus 0.5 μM melatonin for 24 h and during the 0.5 h heat exposure), except that the medium contained unlabeled glutamine. After the treatments, the culture medium was removed, and cells were washed twice with pre-warmed assay buffer. Then, 175 μL of pre-warmed glucose-free, serum-free, and bicarbonate-free Seahorse XF assay medium was added to each well, and cells were incubated at 37 °C in a non-CO_2_ incubator for 1 h before the assay. Glycolytic parameters were determined by sequential injection of glucose (10 mM), oligomycin (1 μM), and 2-deoxy-D-glucose (50 mM). ECAR-derived indices of glycolysis, glycolytic capacity, and glycolytic reserve were calculated according to the manufacturer’s protocol after subtraction of non-glycolytic acidification. Glycolysis was defined as the glucose-stimulated ECAR response, glycolytic capacity as the maximal ECAR response after oligomycin injection, and glycolytic reserve as the difference between glycolytic capacity and glycolysis. These parameters were used as indirect indicators of glycolysis-associated extracellular acidification rather than direct measurements of glycolytic flux. ECAR values were normalized to protein content. Each group included five technical replicate wells per biological replicate, and experiments were independently repeated using three separate primary Sertoli cell preparations (biological replicates).

### 2.6. Statistical Analysis

All in vitro experiments were performed using three independently isolated primary Sertoli cell preparations from different donor goats, which were treated as biological replicates and statistical units. Within each biological replicate, corresponding technical replicates were included, and the mean value of technical replicates was used for statistical analysis. Data are presented as mean ± standard deviation based on biological replicates unless otherwise indicated. Statistical analysis and graphing were performed using IBM SPSS Statistics 26.0 (IBM Corp., Armonk, NY, USA; https://www.ibm.com/products/spss-statistics; accessed on 1 July 2026) and GraphPad Prism 9.0 (GraphPad Software/Dotmatics, Boston, MA, USA; https://www.graphpad.com; accessed on 1 July 2026). Because the number of biological replicates was limited (*n* = 3), formal tests of normality and homogeneity of variance were interpreted cautiously. Biological replicates, rather than technical replicates, were treated as the statistical units. For one-way ANOVA, the statistical model was Y_ij_ = μ + T_i_ + ε_ij_, where Y_ij_ represents the response variable, μ represents the overall mean, T_i_ represents the fixed effect of treatment, and ε_ij_ represents the residual error. Treatment was considered the main fixed effect. No additional covariates or noise variables were included in the model. For endpoints showing no obvious violation of parametric assumptions, comparisons among groups were performed using one-way analysis of variance followed by Tukey’s multiple comparison test. When parametric assumptions were not considered appropriate, the Kruskal–Wallis test followed by Dunn’s post hoc test with Bonferroni correction was used. Given the exploratory nature of the metabolite-level and readout-level analyses, statistical results were interpreted together with effect direction, consistency across biological replicates, and biological plausibility. A value of *p* < 0.05 was considered statistically significant. No additional correction across different metabolite endpoints was applied; therefore, metabolite-level comparisons should be interpreted as exploratory. For the real-time ECAR profile, treatment, time, and the treatment × time interaction were evaluated using two-way repeated-measures ANOVA, with treatment as the between-subject factor and time as the repeated-measures factor. Biological replicate means were used as the statistical units. When a significant interaction or main treatment effect was detected, post hoc comparisons were performed using Tukey’s multiple comparison test. The treatment × time *p* value is reported in the corresponding figure legend.

## 3. Results

### 3.1. Acute Heat Stress Was Associated with Altered Citrate/Cis-Aconitate ^13^C Labeling

**Figure 1:**
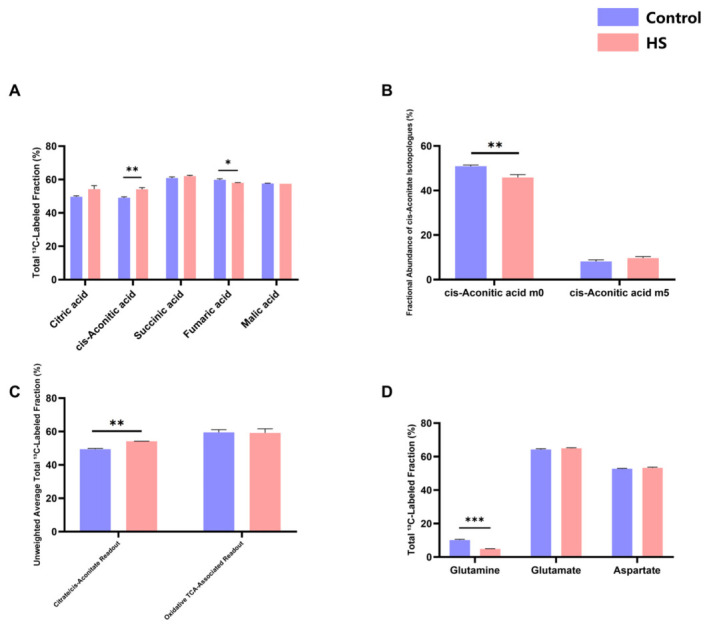
Acute heat stress alters glutamine-derived ^13^C labeling in TCA cycle-related metabolites in primary goat Sertoli cells. (**A**) Total ^13^C-labeled fractions of TCA cycle-related metabolites. (**B**) Mass isotopologue distribution of cis-aconitate. (**C**) Unweighted average total ^13^C-labeled fractions of oxidative TCA-associated metabolites and citrate/cis-aconitate readouts. (**D**) Total ^13^C-labeled fractions of intracellular glutamine and related downstream metabolites. Data are presented as mean ± SD (*n* = 3 biological replicates). * *p* < 0.05, ** *p* < 0.01, and *** *p* < 0.001 indicate statistically significant differences compared with the control group.

**Figure 2:**
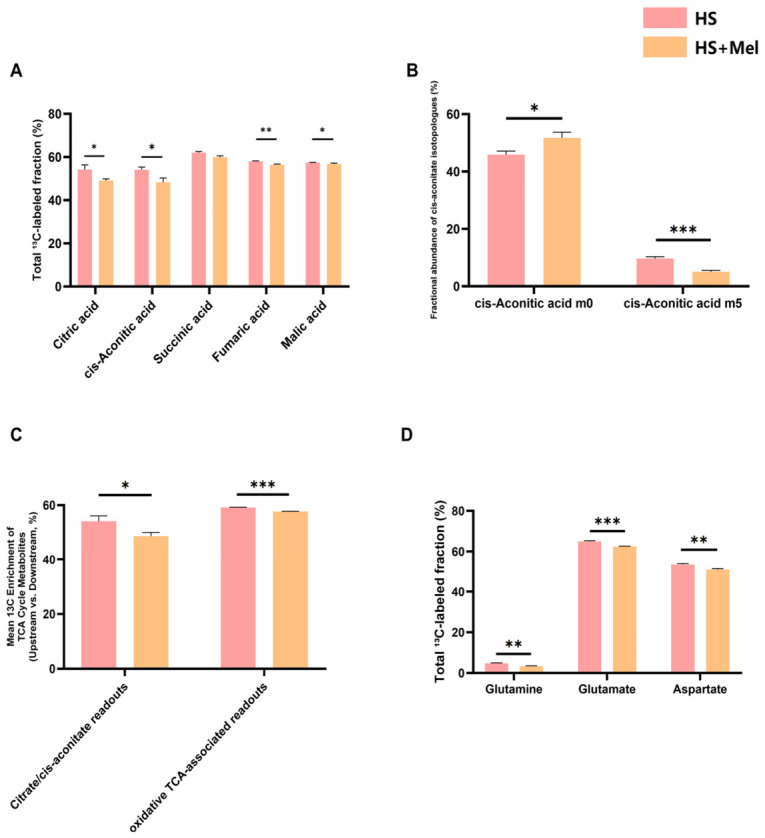
Melatonin attenuates heat stress-associated changes in glutamine-derived citrate/cis-aconitate ^13^C labeling. (**A**) Total ^13^C-labeled fractions of TCA cycle-related metabolites. (**B**) Mass isotopologue distribution of cis-aconitate. (**C**) Unweighted average total ^13^C-labeled fractions of oxidative TCA-associated metabolites and citrate/cis-aconitate readouts. (**D**) Total ^13^C-labeled fractions of glutamine metabolism-related core nodes. Data are presented as mean ± SD (*n* = 3 biological replicates). * *p* < 0.05, ** *p* < 0.01, and *** *p* < 0.001 indicate statistically significant differences compared with the heat stress group.

The statistical comparisons in Figure 1 and Figure 2 were designed according to the specific purpose of each analysis. Figure 1 focuses on the effect of acute heat stress by comparing the heat stress group with the control group, whereas Figure 2 focuses on the modulatory effect of melatonin by comparing the heat stress plus melatonin group with the heat stress group. To determine whether acute heat stress affects glutamine-derived ^13^C-labeling distribution in goat Sertoli cells, endpoint [U-^13^C_5_] glutamine tracing was performed after 24 h of labeling followed by 0.5 h of acute heat exposure. The total ^13^C-labeled fractions and mass isotopologue distributions of selected TCA cycle-related metabolites, including citrate, cis-aconitate, succinate, fumarate, and malate, were analyzed.

Compared with the control group, heat stress significantly increased the total ^13^C-labeled fraction of cis-aconitate (*p* < 0.01), whereas the total ^13^C-labeled fraction of citrate was not significantly changed. The fractional abundance of unlabeled cis-aconitate M + 0 was significantly decreased in the heat stress group (*p* < 0.01). In contrast, the fractional abundance of cis-aconitate M + 5 was not significantly changed (*p* = 0.0588). These results indicate that the increase in cis-aconitate total labeling was not attributable to a statistically significant change in the M + 5 isotopologue alone.

Exploratory readout-group analysis further showed that the unweighted average total ^13^C-labeled fraction of the citrate/cis-aconitate readout group increased from 49.43% in the control group to 54.18% in the heat stress group (*p* < 0.01). In contrast, the average total ^13^C-labeled fraction of oxidative TCA-associated readouts showed no heat stress-associated increase, but rather a slight decrease from 59.47% to 59.15% (*p* = 0.0110). Specifically, the total ^13^C-labeled fractions of succinate and malate were not significantly changed, whereas fumarate showed a 3.06% relative decrease compared with the control value (*p* < 0.05).

The intracellular total ^13^C-labeled fraction of glutamine was markedly reduced in the heat stress group by 52.51% relative to the control group (*p* < 0.001), whereas the total ^13^C-labeled fractions of glutamate and aspartate were not significantly increased.

### 3.2. Melatonin Attenuated Heat Stress-Associated Citrate/Cis-Aconitate ^13^C-Labeling Changes

We next examined whether melatonin pretreatment/co-treatment modulated heat stress-associated changes in glutamine-derived ^13^C labeling. Compared with the heat stress group, melatonin pretreatment/co-treatment reduced the total ^13^C-labeled fractions of cis-aconitate and citrate, with relative decreases of 10.82% and 9.44%, respectively (both *p* < 0.05).

This reduction was most pronounced for the cis-aconitate M + 5 isotopologue. The relative abundance of cis-aconitate M + 5 was reduced by 47.47% in the heat stress plus melatonin group compared with the heat stress group (*p* < 0.001), whereas the cis-aconitate M + 0 fraction was significantly increased by 12.78% (*p* = 0.013).

At the exploratory readout-group level, melatonin significantly reduced the unweighted average total ^13^C-labeled fraction of the citrate/cis-aconitate readout group by 10.13% compared with the heat stress group (*p* < 0.05). The succinate/fumarate/malate oxidative TCA-associated readout group also showed a 2.47% decrease in the unweighted average total ^13^C-labeled fraction after melatonin treatment (*p* < 0.001), although the relative decrease was larger in the citrate/cis-aconitate readout group.

Compared with the heat stress group, the heat stress plus melatonin group also showed lower total ^13^C-labeled fractions of glutamine, glutamate, and aspartate, with relative decreases of 30.26% (*p* < 0.01), 3.81% (*p* < 0.001), and 4.59% (*p* < 0.01), respectively. These changes were interpreted as alterations in relative labeling distributions rather than direct evidence of reduced substrate utilization.

### 3.3. Melatonin Attenuated Heat Stress-Associated ECAR-Derived Acidification

**Figure 3:**
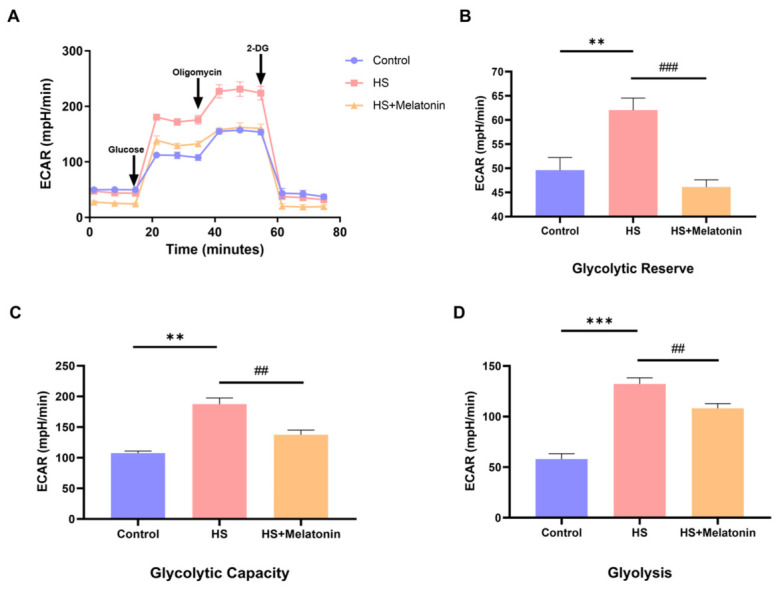
Melatonin attenuates heat stress-associated ECAR-derived glycolysis-associated extracellular acidification. (**A**) Real-time ECAR profiles after sequential injection of glucose, oligomycin, and 2-deoxy-D-glucose. (**B**) Quantitative analysis of glycolytic reserve. For the real-time ECAR profile in (**A**), two-way repeated-measures ANOVA showed a significant treatment × time interaction: F(22, 66) = 61.69, *p* < 0.0001. (**C**) Quantitative analysis of glycolytic capacity. (**D**) Quantitative analysis of basal glycolysis. Data are presented as mean ± SD (*n* = 3 biological replicates). ** *p* < 0.01, and *** *p* < 0.001 indicate statistically significant differences compared with the control group; ## *p* < 0.01 and ### *p* < 0.001 indicate statistically significant differences compared with the heat stress group.

To determine whether changes in glutamine-derived ^13^C labeling were accompanied by altered ECAR-derived glycolysis-associated extracellular acidification, ECAR was measured using a Seahorse glycolysis stress test (Figure 3A).

Compared with the control group, the heat stress group showed significantly higher protein-normalized ECAR-derived glycolysis (*p* < 0.001), glycolytic capacity (*p* < 0.01), and glycolytic reserve (*p* < 0.01) (Figure 3B–D). These results indicate that acute heat stress increased ECAR-derived acidification in primary goat Sertoli cells.

Melatonin pretreatment/co-treatment significantly reduced the heat stress-associated increase in ECAR-derived glycolytic parameters. ECAR-derived glycolysis (*p* < 0.01), glycolytic capacity (*p* < 0.01), and glycolytic reserve (*p* < 0.001) were all reduced in the heat stress plus melatonin group compared with the heat stress group, with values shifting toward those observed in the control group (Figure 3B–D).

## 4. Discussion

In this brief report, endpoint [U-^13^C_5_] glutamine tracing and ECAR analysis were used to examine whether acute heat stress alters central carbon labeling patterns in primary goat Sertoli cells and whether melatonin modulates these responses. The main findings were that acute heat stress increased citrate/cis-aconitate total ^13^C-labeled-fraction readouts, mainly driven by a significant increase in cis-aconitate labeling, whereas it did not uniformly increase oxidative TCA-associated readouts. In parallel, heat stress enhanced ECAR-derived glycolysis-associated extracellular acidification. Melatonin attenuated both the heat stress-associated citrate/cis-aconitate labeling signature and the ECAR-derived acidification response. These findings support our hypothesis that acute heat stress is associated with altered glutamine-derived ^13^C labeling in citrate/cis-aconitate readouts and that melatonin attenuates this stress-associated labeling response. Thus, the study objective was met by demonstrating both the heat stress-associated metabolic labeling alteration and the melatonin-associated attenuation of this response.

The increase in citrate/cis-aconitate total ^13^C-labeled-fraction readouts under heat stress suggests a stress-associated redistribution of glutamine-derived carbon labeling. Because [U-^13^C_5_] glutamine enters central carbon metabolism through glutamate and α-ketoglutarate, citrate and cis-aconitate labeling may be shaped by both oxidative TCA cycling and reductive carboxylation-associated routes [18]. In the present study, the heat stress-associated increase was more evident in cis-aconitate total labeling than in citrate total labeling, and cis-aconitate M + 5 showed only a non-significant numerical increase. Therefore, these data should be interpreted as evidence of altered relative isotope-labeling distribution rather than definitive evidence of increased reductive carboxylation flux.

The decrease in intracellular glutamine total ^13^C-labeled fraction after heat stress further supports the need for cautious interpretation. Changes in glutamine labeling may reflect altered glutamine uptake, intracellular glutamine turnover, precursor-pool labeling, isotope dilution, or exchange between glutamine-derived labeled pools and TCA cycle intermediates. Because the present analysis was based on endpoint mass isotopologue distributions and total labeled fractions, it cannot directly quantify the absolute glutamine utilization rate or pathway flux. Dynamic isotope tracing, precursor-pool normalization, substrate uptake measurements, and metabolic flux modeling will be required to define whether heat stress truly increases reductive carboxylation flux or instead alters labeling through changes in precursor-pool dynamics.

Melatonin attenuated the heat stress-associated citrate/cis-aconitate labeling signature. This finding is notable because melatonin was not accompanied by a uniform increase in oxidative TCA-associated glutamine-derived labeling under the present experimental conditions. Instead, melatonin reduced the total ^13^C-labeled fractions of citrate and cis-aconitate, with a particularly strong decrease in the cis-aconitate M + 5 isotopologue. These results suggest that melatonin may attenuate heat stress-associated metabolic labeling changes rather than merely promoting glutamine-derived oxidative TCA labeling. This interpretation is consistent with the broader view that melatonin can modulate stress-associated metabolic responses in addition to its antioxidant and mitochondria-protective properties [19].

The ECAR data provide a complementary extracellular acidification readout. Acute heat stress increased ECAR-derived glycolysis, glycolytic capacity, and glycolytic reserve, indicating increased glycolysis-associated extracellular acidification rather than directly measured glycolytic flux. Melatonin reduced all three ECAR-derived parameters under heat stress. Together with the isotope-tracing results, these findings suggest that heat stress is associated with coordinated changes in glutamine-derived labeling and glycolysis-associated acidification, both of which are attenuated by melatonin. However, ECAR alone does not directly measure glycolytic flux, and the present study did not include mitochondrial oxygen consumption rate, redox status, or ATP production measurements. Therefore, the ECAR findings should be interpreted specifically as evidence of altered extracellular acidification rather than as direct evidence of restored mitochondrial respiration.

Based on these findings, we propose a working model in which acute heat stress induces central-carbon isotope-labeling remodeling in primary goat Sertoli cells. Under basal conditions, glutamine-derived α-ketoglutarate contributes to TCA-related isotopologue patterns through oxidative TCA-associated routes and routes potentially associated with reductive carboxylation. Under acute heat stress, Sertoli cells exhibit increased citrate/cis-aconitate total-labeled-fraction readouts together with increased ECAR-derived glycolysis-associated acidification. Melatonin attenuates both responses, suggesting that it may reduce stress-associated metabolic labeling remodeling in Sertoli cells.

Several limitations should be acknowledged. First, this study was performed in primary cultured goat Sertoli cells, which cannot fully reproduce the cellular interactions, endocrine regulation, and tissue microenvironment of the testis in vivo. Second, the endpoint isotope tracing strategy used here reflects relative mass isotopologue distributions and total ^13^C-labeled fractions, but does not provide dynamic or absolute metabolic flux information. Third, only glutamine-derived ^13^C-labeling distribution and ECAR-derived acidification parameters were analyzed; other substrates, including glucose, fatty acids, and branched-chain amino acids, may also contribute to heat stress-associated metabolic adaptation. Fourth, the absence of a melatonin-only control group limits the ability to distinguish whether the observed metabolic effects are specific to the interaction between melatonin and heat stress or reflect melatonin-induced metabolic changes independent of heat stress. These limitations indicate that the present results should be interpreted as relative isotope-labeling and ECAR-derived readouts rather than direct evidence of absolute metabolic flux or complete restoration of Sertoli cell metabolic function by melatonin.

Overall, these results support the view that acute heat stress is associated with relative glutamine-derived isotope-labeling redistribution involving citrate/cis-aconitate readouts and increased ECAR-derived acidification in primary goat Sertoli cells. Melatonin attenuates both responses, suggesting that its protective effects under heat stress may involve modulation of nutrient-derived isotope-labeling patterns and glycolysis-associated acidification.

## 5. Conclusions

In conclusion, acute heat stress altered glutamine-derived ^13^C-labeling patterns in primary goat Sertoli cells, primarily reflected by increased cis-aconitate labeling and an elevated citrate/cis-aconitate total-labeled-fraction readout. These endpoint labeling patterns are compatible with altered citrate/cis-aconitate labeling potentially related to reductive carboxylation but do not directly demonstrate altered metabolic flux. Melatonin pre-/co-treatment attenuated the heat stress-associated labeling signature and reduced ECAR-derived extracellular acidification, suggesting that melatonin modulates stress-associated metabolic readouts in Sertoli cells.

## Data Availability

The original contributions presented in this study are included in the article. Further inquiries can be directed to the corresponding authors.
